# Prevalence and predictors of occupational asthma among workers in detergent and cleaning products industry and its impact on quality of life in El Asher Men Ramadan, Egypt

**DOI:** 10.1007/s11356-022-18558-8

**Published:** 2022-01-15

**Authors:** Amani Shawki Ahmed, Dalia Anas Ibrahim, Tarek Hamdy Hassan, Wael Galal Abd-El-Azem

**Affiliations:** 1 Community, Environmental and Occupational Medicine Department, Faculty of Medicine, Zagazig University, Zagazig, Egypt; 2 Chest Department, Faculty of Medicine, Zagazig University, Zagazig, Egypt

**Keywords:** Occupational asthma, Detergent, Cleaning products, Asthma-specific quality of life

## Abstract

Cleaning products are mixtures of many chemical ingredients that are known to contain sensitizers, disinfectants, and fragrances, as well as strong airway irritants which associated with lower respiratory tract and asthma symptoms.

The aim of this study is to assess the prevalence and possible risk factors of occupational asthma and its effect on quality of life among workers in detergent and cleaning products industries in El Asher men Ramadan city.

This cross-sectional study was conducted on 780 workers. All participants were personally interviewed at their workplaces and were subjected to a questionnaire regarding sociodemographic, work characteristics and asthma symptoms, clinical examination, chest X-ray, spirometer, and bronchodilator test.

The prevalence of occupational asthma among the studied workers was 35.4%. Multivariate logistic regression analysis revealed that female gender [odds ratio 1.397; 95% CI 1.09–1.96], manually working participants [odds ratio 3.067; 95% CI 1.72–5.46], and history of atopy [odds ratio 1.596; 95% CI 1.09–2.33] were risk factors for development of occupational asthma. The total mean score of asthma-specific quality of life was significantly lower in asthmatic (5.10 ± 0.49) than non-asthmatic workers (5.89 ± 0.46) (P < 0.01) indicating impairment of quality of life among asthmatic group.

Workers in detergent and cleaning products industry are at higher risk for developing occupational asthma that adversely affects their general health and quality of life.

## Introduction

The respiratory health problems have been reported in workers exposed to a variety of occupational exposures during their work processing (Baur and Bakehe, 2014). Respiratory disorders caused by occupational exposures are influenced by type of offending dust, dose, duration of exposure, and genetic factors (Subbarao et al., 2009).

Chemical substances used in cleaning products including enzymes and powder which are respiratory sensitizers are known to cause occupational asthma among worker. It is also an irritant and may give rise to short-term or long-term respiratory disorder up to chronic bronchitis (Dykewicz, 2009; Moscato et al., 2012).

Previous studies on assessing occupational asthma have shown that the proportion of adult asthma cases attributable to occupational exposure is between 10 and 15% (Mwanga & Jeebhay, 2013), while the prevalence of occupational asthma associated with exposure to cleaning products was 12% of the confirmed cases of work-related asthma (Rosenman et al. 2003).

The oxidative stress response has recently been linked to asthma in cleaners who work in hazardous environments (Folletti et al., 2017). Radon et al. (2016) reported that the increased asthma frequency in cleaners could be explained by poor psychosocial working conditions and a stress-induced inflammatory response mechanism. Furthermore, early life exposure appears to have a role in the airway vulnerability of cleaners exposed later in life (Svanes et al., 2015).

In order to limit the risk of occupational allergy and asthma, guidelines for the safe handling of enzymes have been established in the detergent sector (Adisesh et al, 2011). Prospective surveillance for the development of enzyme-specific IgE antibody before the emergence of allergic symptoms is essential to the efficacy of the management of enzyme-induced allergy and asthma (Sarlo et al., 2010).

Asthma is frequently associated with psychiatric disorders such as mood and anxiety disorders, and individuals with depressive disorders have been demonstrated to have worse asthma control and a poor quality of life (QOL) (Eisner et al., 2005; Lavoie et al., 2009). Patients with psychiatric comorbidity tend to use more health care than those without it (Richardson et al., 2008).

Coverage and researches for occupational asthma were limited in Egypt especially among workers in detergent and cleaning products industry and its impact on quality in life. The current study aimed to assess the prevalence and possible risk factors of occupational asthma and its effect on quality of life among workers in detergent and cleaning products industries in El Asher men Ramadan city.

## Subjects and method

### Study population

This cross-sectional study was conducted on workers in detergent and cleaning product factories in El Asher men Ramadan city. Data were collected during 6 months from start of May 2020 to the end October 2020.

### Sample size and sampling technique

the prevalence of occupational asthma associated with cleaning products exposure was (12%) of the confirmed cases of work-related asthma (Rosenman et al. 2003) so sample size calculated by Epi6 program to be 711 with 10% non-responder rate so sample was 780 with power of study 80% and confidence level 95.0%. Multi-stage sampling technique was used for selection of participants. Two factories were selected, and then attendance sheet of workers was used to select the participant from each factory.

Inclusion criteria: male and female workers who were working at least for 1 year aged from 18 to 60 years old.

Exclusion criteria: workers with history of respiratory problems before joining their current work and who refuse to participate in the study.

### Study procedures

Each participant was subjected to the following:

- 1-A semi-structured questionnaire was used depending on previous studies regarding sociodemographic, work characteristics, and asthma symptoms:
-Age, sex, smoking, occupational process, PPE, and duration
-Symptoms (cough, dyspnea, wheeze), symptoms resolve after work or in holidays or not, completely or partially trigger of asthma, asthma symptoms leading to night awaking, family history of atopy, childhood asthma, occupational periodic examination, diagnosed as occupational asthma, work replacement or compensation for occupational asthma (Nicholsonet al., 2001; Ladics et al., 2014; Porsbjerg et al., 2018).
- 2-Clinical examination.
- 3-Chest X-ray: if indicated to exclude other lesions. Portable X-ray machine was used in the workplace.
- 4-Spirometer: According to GINA guidelines (Reddel et al., 2015), participants were asked to perform forced expiratory maneuver where three reproducible curves were obtained (± 5%). The spirometry was done from 8 to 11 am. The measured parameters included FVC, FEV1, FEV1%, and PEFR.
- 5-Bronchodilator test: the test was done for all participants.

All examinations were done at the workplace in a special room in each factory used during the period of the field study.

Considerations for conducting spirometry during COVID-19 (CDC 2020):

-Screen the workers before conducting the spirometry test: by taking their temperature and examining for potential COVID-19 symptoms such as chills, cough, sore throat, loss of taste or smell, runny nose, and nasal congestion.
-Staff used N95 mask and face shield.
-All workers used a face mask.
-Seventy percent alcohol was available for washing hands (for workers and staff) prior to and at the end of spirometry.
-Single-use nose clips and mouthpieces were used.
-Disinfection procedures were conducted after every worker.

### *Asthma Quality of Life Questionnaire (AQLQ) (*Juniper et al., 1999*)*

The questionnaire includes 32 items that assess asthma quality of life through the principal four domains which may be negatively affected by asthma.

-Asthma symptoms
-Activity limitations
-Emotional function
-Exposure to environmental stimuli

Every question is scored from 1 (severe impairment) to 7 (no impairment), and the total score is the mean of these four scores.

### Statistical analysis

Data collected were coded and entered using Microsoft Excel software and Statistical Package for the Social Sciences (SPSS version 20.0) used for analysis. Qualitative data was represented as number and percentage, quantitative continues group by mean ± SD, testing association and differences of qualitative data by Chi square test (X2), while differences between quantitative independent groups by t test. Multiple logistic regression analysis was carried out to identify independent risk factors of occupational asthma. *P*-value was significant at < 0.05 for significant results.

## Results

Most of the included participants were males (76.2%); the participants’ age ranged from 21 to 56 years with mean age of 36.43 years, and mean duration of work was 7.79 years. 88.2% of them were manual workers, and 11.8% were working as observers. Majority of participants were married (74.7%), and 24% were smokers (all smokers were males). The history of atopy was positive in 17.7% of our studied group, while only 7.8% of them is the use of protective measures as shown in Table 1.

**Table 1:** Sociodemographic and work characteristics among studied worker of detergent and cleaning products (*N* = 780)

Age (years)	Mean ± SD	36.43 ± 9.35	
	**Median (range)**	**35.0 (21–56)**	
**Duration of work (years)**	**Mean ± SD**	**7.79 ± 3.86**	
	**Median (range)**	**9.0 (1–25)**	
		**N**	**%**
**Sex**	**Female**	**186**	**23.8**
	**Male**	**594**	**76.2**
**Occupation**	**Manual**	**688**	**88.2**
	**Observer**	**92**	**11.8**
**Marital state**	**Married**	**583**	**74.7**
	**Single**	**182**	**23.3**
	**Widow**	**15**	**1.9**
**History of atopy**	** − VE**	**642**	**82.3**
	** + VE**	**138**	**17.7**
**Smoking**	**No**	**593**	**76**
	**Smoker**	**187**	**24**
**Use of protective measures**	**No**	**719**	**92.2**
	**Yes**	**61**	**7.8**

All smokers were males; no periodic medical examination for workers was done.

The prevalence of occupational asthma among the studied workers was 35.4%. 23.7% of participants were previously diagnosed as occupational asthma, while the new cases were 11.7% of participants as shown in Table 2.

**Table 2:** Prevalence of occupational asthma among the studied workers

	**No (780)**	**%**
**Prevalence of occupational asthma**	276	35.4
Workers previously diagnosed with occupational asthma	185	23.7
Workers newly diagnosed with occupational asthma	91	11.7
**Workers free of occupational asthma**	504	64.6

**Fig. 1:**
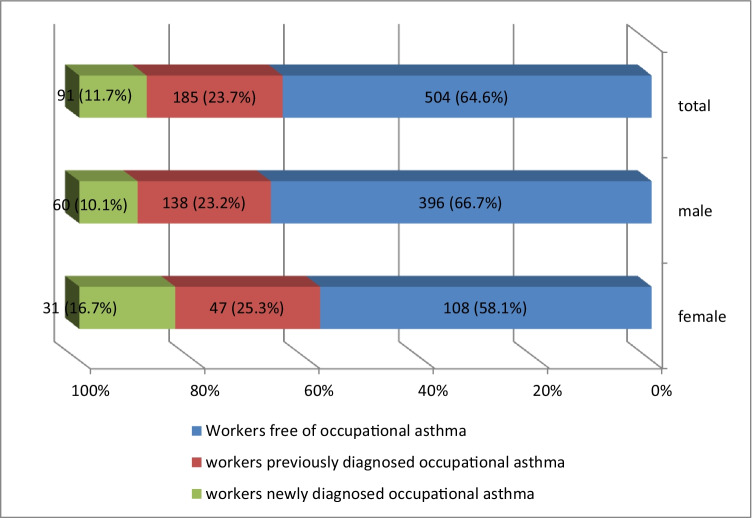
Gender distribution of occupational asthma among the studied workers.Note: *X*^2^ = 4.58, *P*-value = 0.032 (statistical significant difference)

The occupational asthma was prevalent among female workers; 25.3% of females were previously diagnosed with OA compared to 23.2% of males, while 16.7% of females were newly diagnosed with OA compared to 10.1% of males with statistically significant difference (*p* < 0.05) as shown in Fig. 1.

According to Gina guidelines, 58.4% of workers who were previously diagnosed with occupational asthma were uncontrolled. FEV1/FVC was normal in 49.7%, whereas 42.1% reported obstructive and 8.1% reported obstructive with mild restrictive symptoms. Regarding FEV1, 49.7% were normal, while 41.6% and 8.6% had mild and moderate symptoms, respectively. 66.5% of these workers were on long-term treatment, and 82.7% of them changed their nature of work due to asthma. 50.3% of them had other allergy symptoms such as allergic rhinitis (Table 3).

**Table 3:** Clinical characters of the previously diagnosed workers with occupational asthma (*N* = 185)

Duration	Mean ± SD	6.45 ± 2.26	
	**Median (Range)**	**5.0 (2–12)**	
		**N**	**%**
**Control of asthma according to Gina guidelines**	**Controlled**	**77**	**41.6**
	**Uncontrolled**	**108**	**58.4**
**FEV1/FVC**	**Normal**	**92**	**49.7**
	**Obstructive**	**78**	**42.1**
	**Obstructive with mild restrictive**	**15**	**8.1**
**FEV1**	**Normal**	**92**	**49.7**
	**Mild**	**77**	**41.6**
	**Moderate**	**16**	**8.6**
**Long-term treatment**	**No**	**123**	**66.5**
	**YES**	**62**	**33.5**
**Change work due to asthma**	**No**	**153**	**82.7**
	**Yes**	**32**	**17.3**
**Other site of allergy**	**No**	**92**	**49.7**
	**Allergic rhinitis**	**93**	**50.3**

Manually working and positive history of atopy were significantly associated with development of occupational asthma, while age, duration of work, smoking, and use of protective measure were not significantly associated with development of occupational asthma among our studied participants as shown in Table 4.

**Table 4:** Association between sociodemographic and work characteristics and occupational asthma among the studied worker of detergent and cleaning products

		**Workers without occupational asthma (*N* = 504)**	**Workers with occupational asthma (*N* = 276)**	**Sig. test**	***P* value**
**Age (years)**		36.17 ± 9.22	36.92 ± 9.57	*t* = 1.071	0.285
**Duration of work (years)**		8.03 ± 2.96	7.76 ± 2.38	*t* = 1.718	0.091
**Occupation**	**Manual**	427 (84.7%)	261 (94.6%)	*X*^2^ = 16.60	< 0.001**
	**Observer**	77 (15.3%	15 (5.4%)		
**History of atopy**	**Negative**	427 (84.7%)	215 (77.9%)	*X*^2^ = 5.71	0.017*
	**Positive**	77 (15.3%)	61 (22.1%)		
**Smoking**	**Non-smoker**	374 (74.2%)	219 (79.3%)	*X*^2^ = 2.58	0.108
	**Smoker**	130 (25.8%)	57 (20.7%)		
**Use of protective measure**	**No**	468 (92.9%)	251 (90.9%)	*X*^2^ = 0.90	0.34
	Yes	36 (7.1%)	25 (9.1%)		

All smokers were males.

*Statistically significant (*P* < 0.05).

**Highly statistically significant (*P* < 0.001).

Multivariate logistic regression analysis revealed that female gender [odds ratio 1.397; 95% CI 1.09–1.96], manually working participants [odds ratio 3.067; 95% CI 1.72–5.46], and history of atopy [odds ratio 1.596; 95% CI 1.09–2.33] were risk factors for development of occupational asthma among worker in detergent and cleaning products as shown in Table 5.

**Table 5:** Multivariate logistic regression analysis of risk factors associated with occupational asthma among the studied worker of detergent and cleaning products

Risk factors	B	S.E	Wald	Significance	OR	95% C.I OR	
						**LL**	**UL**
**Positive history of atopy**	0.467	0.194	5.814	0.016*	1.596	1.091	2.33
**Manually working**	1.121	0.294	14.512	0.000**	3.067	1.723	5.46
**Female gender**	0.434	0.175	5.662	0.047*	1.397	1.092	1.96

*LL* lower limit, *UL* upper limit.

*Statistically significant (*P* < 0.05).

**Highly statistically significant (*P* < 0.001).

The average scores of the components of asthma-specific quality of life showed the lower score (indicating higher difficulty), while the higher score (indicating better quality). On comparing these components between asthmatic and non-asthmatic workers, among asthmatic workers, the mean score of symptoms (4.52 ± 0.84), activity limitations (4.53 ± 0.86), and exposure to environmental stimuli (5.78 ± 0.49) were lower than that of non-asthmatic (5.94 ± 0.51, 6.07 ± 0.60, and 5.93 ± 0.66, respectively) with statistically significant difference (*P* < 0.01), while there is no statistical difference between them as regarding emotional function. The total mean score of asthma-specific quality of life was significantly lower in asthmatic (5.10 ± 0.49) than non-asthmatic workers (5.89 ± 0.46) (*P* < 0.01) as shown in Table 6.

**Table 6:** Quality of life between workers with occupational asthma and others without occupational asthma

	Workers without occupational asthma (*N* = 504)	Workers with occupational asthma (*N* = 276)	*t*	*p*
**Symptoms**				
Mean ± SD	5.94 ± 0.51	4.52 ± 0.84		
Range (max–min)	3 (4–7)	5 (2–7)	25.47	0.000**
**Activity limitations**				
Mean ± SD	6.07 ± 0.60	4.53 ± 0.86		
Range (max–min)	4 (3–7)	5 (2–7)	26.33	0.000**
**Emotional function**				
Mean ± SD	5.60 ± 0.94	5.56 ± 0.96		
Range (max–min)	3 (4–7)	3 (4–7)	0.53	0.59
**Exposure to environmental stimuli**				
Mean ± SD	5.93 ± 0.66	5.78 ± 0.49		
Range (max–min)	3 (4–7)	3 (3–6)	3.44	0.001*
**Total**				
Mean ± SD	5.89 ± 0.46	5.10 ± 0.49		
Range (max–min)	2.5 (4.5–7)	2.50 (3.75–6.25)	21.6	0.000**

*Statistically significant (*P* < 0.05).

**Highly statistically significant (*P* < 0.001).

## Discussion

Workers in detergent and cleaning products industry are vulnerable group for developing occupational asthma and other respiratory problems as they are exposed to a wide range of irritants and sensitizers in the chemical substances used, besides common indoor allergens and pollutants (Quirce & Barranco, 2010).

It is essential to get an appropriate diagnosis of occupational asthma in workers. The disorder has serious health effects for affected individuals, as well as major socioeconomic consequences for both workers and employers. Missing an OA diagnosis can lead to extended exposure to a causal agent and disease progression (Vandenplas et al., 2017).

In the present study, the diagnosis of OA was based on GINA guidelines including a history taking of exposure, respiratory symptoms and clinical examinations, spirometry, chest X-ray, and bronchodilator test. Our study revealed that 35.4% was the prevalence of occupational asthma; 23.7% of studied workers were previously diagnosed, while the new cases were 11.7%. The high prevalence of occupational asthma among this workers are explained by that cleaning agents and detergents contain irritants like chlorine and ammonia, as well as possible sensitizers including quaternary ammonium compounds and fragrances (Dumas and Moual, 2016).

Vandenplas et al. (2013) conducted a retrospective case series of workers with work-related asthma who worked in the cleaning products industry. Thirty-nine percent of the workers were diagnosed positive for asthma caused by cleaning agents. Bleach was the most frequently attributed cleaning agent with asthma symptoms (Vandenplas et al., 2013).

Both allergic and irritating processes are implicated in asthma development caused by cleaning chemicals and disinfectants; however, the irritant mechanisms are the most prominent (Siracusa et al. 2013; Matteis and Cullinan, 2015). When the airway epithelium is damaged as a result of repeated irritating exposure, the inflammatory Th2 response is triggered (Tarlo & Lemiere, 2014).

Several sociodemographic and work characteristics are associated with increased risk of developing occupational asthma. In the present study, female gender was significantly associated with development of occupational asthma (*p* < 0.05). Similarly, Torén et al. (2011) reported that occupational asthma was shown to be more common in women than it was in men. Cleaning agent exposure, both at home and at work, enhanced the risk (OR 2.0, 95% CI 1.2–3.3) (Le Moual et al., 2013).

In our study, a significant association was noticed between manually working and occupational asthma than observers as manually working has a direct contact with the chemical and hence more exposure rate.

In the present study, positive history of atopy was significantly associated with development of occupational asthma. It may contribute to concurrent exposure to a mixture of enzymes in the detergent industry, and it is likely to have a synergistic effect in augmenting respiratory allergy (Hole et al., 2000). Basketter et al. (2012) reported that surveillance of detergent workers had led them to certain conclusions about the relationship between enzyme exposure, IgE antibody induction, and the elicitation of asthma symptoms (Basketter et al., 2012).

The implementation of exposure control tools resulted in a significant reduction in exposure; this resulted in the elimination of symptoms among IgE-positive workers as well as low rates of new sensitization in the workforce (Larsen et al., (2020). But, in our study, the use PPE was not significantly associated with asthma development as only 7% of our workers used PPE; this explain the high prevalence of occupational asthma among our studied group.

In occupational frameworks, smoking is one of the risk factors responsible to accelerate the process of occupational asthma maturity due to the combination bond of smoking, and occupational irritants exposure is significant for developing OA (Badar et al., 2016). Our study revealed non-significant association between OA and smoking as only 20% of our studied workers were smokers and all of them were males; no smoking among females, so association was difficult to be detected.

Occupational asthma can disrupt a patient’s daily life, interfering with professional, familial, and social activities. It is also associated with a high rate of prolonged work disruption and income loss (Vandenplas and D’Alpaos 2010). Asthma-related QoL refers to the global impact of asthma and its management on physical, emotional, and social aspects of patients’ functioning (Baiardini et al., 2006).

In our study, on comparing the subscales of asthma-related quality of life between asthmatic and non-asthmatic workers, among asthmatic workers, the mean scores of symptoms, activity limitations, and exposure to environmental stimuli were lower than that of non-asthmatic with statistically significant difference (*P* < 0.01) referring to lower quality of life among asthmatic regarding these domains. But there is no statistical difference between them as regarding emotional function. The total mean score of asthma-specific quality of life was significantly lower in asthmatic than non-asthmatic workers (*P* < 0.01).

Malo et al. (1993) revealed a statistically significant difference was observed in all QoL domains (asthma symptoms, emotional dysfunction, limitation of activities, and environmental stimuli) moreover, in the total score of the quality of life. It was also reported that workers with OA, even when occupational exposure was ceased, have a slightly but significantly lower QoL than those without OA.

Miedinger et al. (2011) investigated individuals with OA 2 years after being removed from the causative agent and found that 35% of patients with OA had anxiety problems and 23% had dysthymia (a chronic form of depression). They also reported that patients with OA experienced higher levels of psychological distress and moderately reduced disease-specific QOL.

## Conclusion

Workers in detergent and cleaning products industry are at higher risk for developing occupational asthma. Cleaning products contain chemical substances that could contribute to the development of occupational asthma or exacerbate the symptoms among asthmatics due to respiratory tract irritation or sensitization mechanisms that adversely affect their general health and quality of life. Use of personal protective equipment at work and periodic medical examination could be solutions to protect workers from developing occupational asthma.

## Data Availability

The datasets used and analyzed during this study are available from the corresponding author on reasonable request. **Ethical consideration.** The study protocol was approved by the Institutional Review Board (IRB) of Faculty of Medicine, Zagazig University. Approval from the workplace management team was obtained.
